# Clozapine generates obsessive compulsive disorder-like behavior in mice

**DOI:** 10.1186/s13041-020-00621-5

**Published:** 2020-05-29

**Authors:** Shinwon Kang, Hyun Jong Noh, Soo Hyeon Bae, Yong-Seok Kim, Hogun Lew, Jisoo Lim, Se Joo Kim, Kyung Sue Hong, Jong-Cheol Rah, Chul Hoon Kim

**Affiliations:** 1 Department of Pharmacology, BK21 PLUS Project for Medical Science, Brain Research Institute, Yonsei University College of Medicine, Seoul, 03722 South Korea; 2 Q-fitter, Inc., Seoul, 06591 South Korea; 3 Korea Brain Research Institute, Daegu, 41068 South Korea; 4 Department of Brain and Cognitive Sciences, DGIST, Daegu, 42988 South Korea; 5 Department of Psychiatry, Yonsei University College of Medicine, Seoul, 03722 South Korea; 6 Department of Psychiatry, Sungkyunkwan University School of Medicine, Samsung Medical Center, Seoul, 06351 South Korea; 7 Severance Biomedical Science Institute, Yonsei University College of Medicine, Seoul, 03722 South Korea

## Abstract

Clozapine is thought to induce obsessive compulsive symptoms (OCS) in schizophrenic patients. However, because OCS are often comorbid with schizophrenia regardless of clozapine treatment, it remains unclear whether clozapine can generate OCS de novo. Thus, it has been difficult to establish a causal link between clozapine and OCS in human studies. To address this question, we asked whether chronic treatment with clozapine can induce obsessive compulsive disorder (OCD)-like behavior in mice. We injected mice with long-term continuous release pellets embedded with clozapine four times at 60-day intervals and then monitored the mice for signs of OCD-like behavior up to 40 wk. of age. We found clozapine increases grooming behavior as early as 30 wk. of age. We also investigated the effect clozapine on grooming behavior in *Sapap3* knockout (KO) mice, which are a well-known animal model of OCD. In *Sapap3* heterozygous KO mice, clozapine increases grooming behavior much earlier than in wild-type mice, suggesting a clozapine-OCD gene interaction. Fluoxetine, which is often used in the treatment of OCS and OCD, reduced the grooming behavior induced by clozapine. These data demonstrate that chronic clozapine treatment can generate OCD-like behavior in mice and support the hypothesis that clozapine produces de novo OCS regardless of schizophrenia status.

Antiserotonergic second generations antipsychotics (SGA) like clozapine, risperidone, and olanzapine are widely used in the treatment of schizophrenia. In contrast to other SGA that primarily work on dopamine receptors, they have pronounced antiserotonergic properties [1]. A series of clinical reports have suggested that the antiserotonergic SGA may also increase the occurrence of OCS in patients with schizophrenia [2–4]. But because schizophrenia and OCS are often comorbid, this effect remains controversial. Moreover, even if the antiserotonergic SGA do promote OCS, it is difficult to tell whether this is de novo OCS or simply an unmasking or exacerbation of pre-existing OCS. There are several lines of indirect evidence that support a causal relationship between antiserotonergic SGA and OCS in schizophrenic patients [4]. First, the prevalence of OCS increased after market approval of the first SGA [4]. Second, OCS are more prevalent in schizophrenic patients treated with antiserotonergic SGAs [5, 6]. Third, OCS severity is positively correlated with antiserotonergic SGA treatment duration and dose size [7]. Although these lines of evidence strongly suggest a link, more prospective cohort studies and studies using disease-relevant animal models are warranted to fully address this question.

Here, we used normal mice (B6J) to determine whether clozapine can induce de novo OCD-like symptoms. Clozapine is the antiserotonergic SGA most commonly associated with OCS in schizophrenia. Because the onset time of OCS in schizophrenic patients treated with clozapine is 1 to 96 months (median 19.5 months) [6], we decided to use specially designed clozapine pellets that slowly and continuously release clozapine for 60 days. We treated mice (B6J) with clozapine for 240 days by implanting the first clozapine pellet at 12 wk. of age with follow-up injections at 60-day intervals (Fig. 1a, see Additional file 1 for the detailed methods). When we measured blood concentration of clozapine 10 days after injection, we found evidence of sustained maintenance of plasma clozapine concentrations (see Additional file 2: Figure S1A). In mice at 15, 20, 30, and 40 wk. of age, we recorded 2 h of grooming behaviors using a video camera. Two independent experimenters then manually scored grooming duration and bout number. We found clozapine increases grooming time in wild-type mice beginning at 30 wk. of age (18 wk. after 1st clozapine pellet injection) (Fig. 1b, left). We also found mice treated with clozapine pellets for 40 wks engage in more grooming bouts (Fig. 1b, right). We did not see any change in weight between clozapine- and placebo-treated mice (see Additional file 2: Figure S1B). In human studies, genetic interactions of *SAPAP3* and *SLC1A1* gene variants are predictive of antiserotonergic SGA-induced OC symptoms [8]. The homozygous deletion of *Sapap3* in mice induces grooming behavior that is severe enough to develop facial and neck skin lesions [9]. Thus, we also tested the effect of clozapine on OCD-like behavior in *Sapap3* knockout mice. We started to observe the development of skin lesions from 20 wk. in homozygous mice and this reached 100% penetration by the age of 40–60 wk. Clozapine does not accelerate the development of skin lesions in these *Sapap3* homozygous KO mice, probably because the grooming behavior is already so severe (see Additional file 2: Figure S1C). *Sapap3* heterozygous KO mice, in contrast, do not show increased grooming behavior or skin lesions [9]. Interestingly, we found clozapine induces grooming behavior in *Sapap3* heterozygous KO mice earlier than in wild-type mice (Fig. 1c). We next tested whether fluoxetine, a selective serotonin reuptake inhibitor, can reduce the grooming behavior induced by chronic clozapine treatment. As shown in Fig. 1d, we observed that 10 days of daily intraperitoneal fluoxetine injections into mice administered clozapine for 30 wks significantly reduces their grooming time. This suggests 5-HT signaling is involved in the pathogenesis of clozapine-induced OCS and that OCD-like behavior in mice responds to the therapeutic agents typically used in humans.

**Fig. 1:**
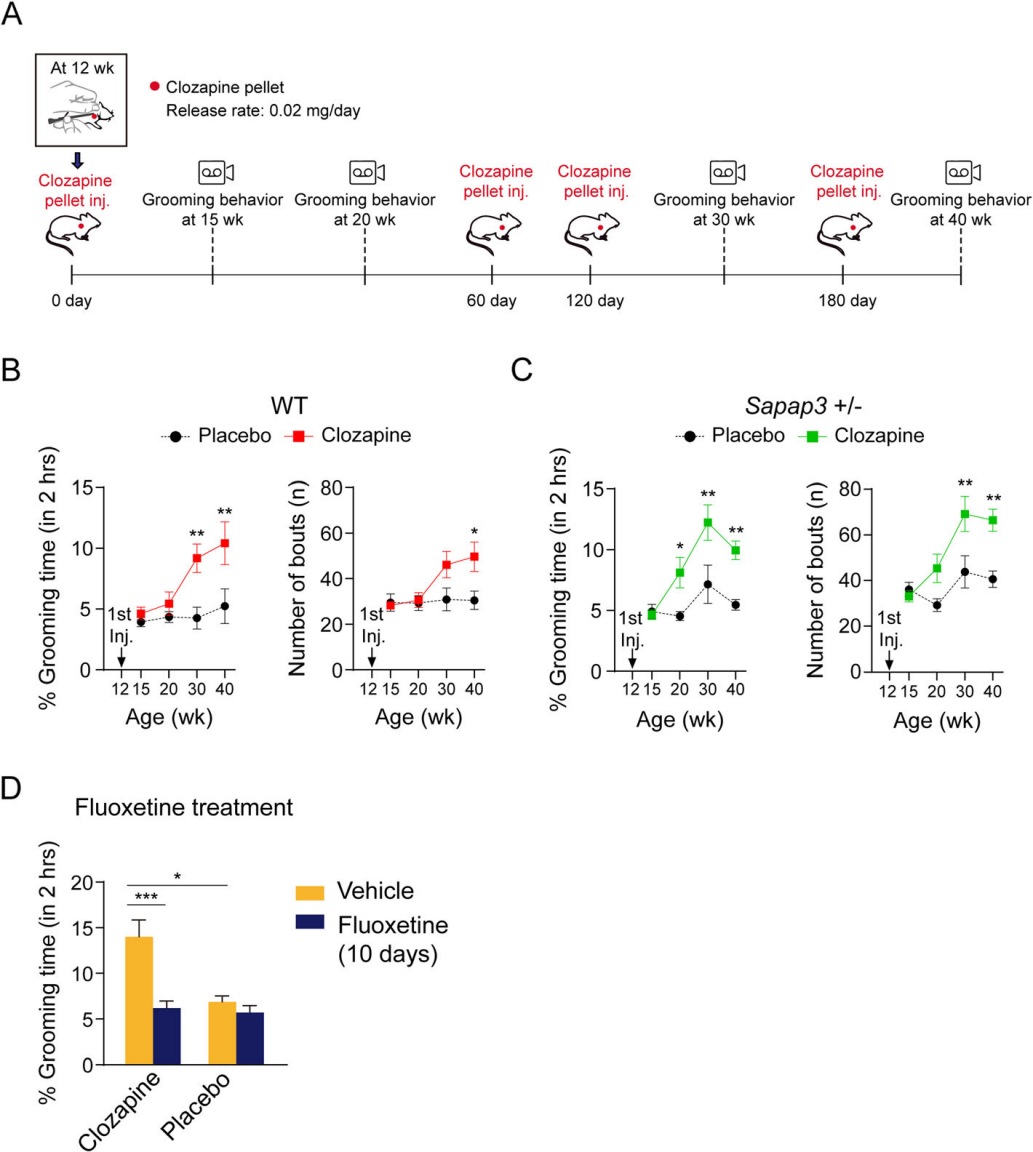
Clozapine-treated mice showed over grooming behavior. **a** Schematic diagram of experimental procedure. Wild-type and *Sapap3*^*+/−*^ adult mice were administered with either clozapine or placebo for 28 wks, starting from age of 12 wk. **b** Clozapine-treated wild-type mice showed increased repetitive grooming behavior. Mice were injected with clozapine or placebo pellets at the age of 12 wk. with 60-day interval pellet injection. Mice behavior was recorded for 2 h and analyzed at the age of 15, 20, 30, and 40 wk. (Two-way RM ANOVA, main effect of treatment; F(1,16) = 9.161, *p* = 0.008, main effect of time; F(3,48) = 5.525, *p* = 0.002, treatment x time interaction; F(3,48) = 2.981, *p* = 0.04, ** *p* < 0.01) (left). Grooming lasted more than 3 s was counted as a bout (Two-way RM ANOVA, main effect of treatment; F(1,16) = 6.073, *p* = 0.025, main effect of time; F(3,48) = 3.491, *p* = 0.023, treatment x time interaction; F(3,48) = 2.674, *p* = 0.058, * *p* < 0.05) (right). **c** Clozapine-treated *Sapap3*^*+/−*^ mice showed increased repetitive grooming behavior earlier than clozapine-treated wild-type mice. *Sapap3*^*+/−*^ mice were injected with clozapine or placebo pellets at the age of 12 wk. with 60-day interval pellet injection. Mice behavior was recorded for 2 h and analyzed at the age of 12, 15, 20, 30 and 40 wk. (Two-way RM ANOVA, main effect of treatment; F(1,12) = 16.99, *p* = 0.01, main effect of time; F(3,36) = 10.12, *p* < 0.001, treatment x time interaction; F(3,36) = 3.348, *p* = 0.03, * *p* < 0.05, ** *p* < 0.01) (left). Numbers of grooming bouts were counted from 2 h video-recording of grooming behavior. Grooming bouts were increased in *Sapap3*^*+/−*^ mice after 18 wk. of clozapine treatment (Two-way RM ANOVA, main effect of treatment; F(1,12) = 14.16, *p* = 0.003, main effect of time; F(3,36) = 11.17, *p* < 0.001, treatment x time interaction; F(3,36) = 4.095, *p* = 0.013, ** *p* < 0.01) (right). **d** Fluoxetine treatment ameliorated overgrooming behavior in clozapine-treated wild-type mice (Two-way ANOVA, main effect of fluoxetine treatment; F(1,6) = 21.75, *p* = 0.003, main effect of clozapine treatment; F(1,6) = 9.27, *p* = 0.023, fluoxetine treatment x clozapine treatment interaction; F(1,6) = 7.071, *p* = 0.038, * *p* < 0.05, *** *p* < 0.001) *n* = 4 per group. All data are presented as means ± SEM

In this study, we demonstrated for the first time that chronic clozapine administration can induce OCD-like behavior in mice. We provide new data on the long-standing question of whether clozapine induces de novo OCS even in normal conditions. There are several reasons this mouse model is useful for the study of clozapine-induced OCS. First, as in humans [6], significant time is required for the induction of OCD-like behavior after clozapine administration in mice. Second, this model meets all the criteria of validity for animal models of psychiatric disorders [10]; the mouse model shows OCD-like behavior (face validity), has clozapine as an inducer (construct validity), and responds to fluoxetine (predictive validity). Lastly, we confirmed in *Sapap3* KO mice a clear interaction between clozapine and a gene linked to clozapine-induced OCS in humans [8].

While clozapine is the only antipsychotic agent indicated for treatment-resistant schizophrenia [1], the occurrence of OCS after initiation of clozapine treatment is associated with additional functional impairment and poor prognosis of the disease [4]. This animal model will provide a new opportunity for researchers trying to develop effective antipsychotics that do not promote OCS as a side effect. This model will also make it easier to study the molecular and circuit mechanisms contributing to OCS from a translational perspective. The therapeutic effect of fluoxetine we observed suggests the involvement of 5-HT receptors (5-HT_1A_, 5-HT_2A_, and 5-HT_2C_), which are thought to be targets of clozapine [1]. The association of *SLC1A1* polymorphism with symptom occurrence in human also hints at a potential role for glutamate neurotransmission [11]. It seems that the effects of glutamate-related genes on clozapine-induced OCS represent a genetic predisposition (or susceptibility) rather than playing a causative role. Fluoxetine also inhibits compulsive grooming behavior of *Sapap3* homozygous KO mice [9], but only when administered over a long period of time. This suggests that acute changes in serotonin levels do not directly suppress compulsive behaviors. Instead, selective serotonin uptake inhibitors may alter clozapine-induced derangement in OCD-related neural substrates like the cortico-striato-thalamo-cortical circuit [12, 13]. The relative importance and contribution of each of these remains unclear. Further study of the actions of clozapine in this new mouse model will improve our understanding of the pathogenesis of OCS and accelerate the development of antipsychotics that lack OCS-inducing side effects.

## Supplementary information

**Additional file 1.** Raw data of the tested parameters in wild-type.

**Additional file 2: Figure S1.** A Plasma clozapine concentration 10 days after clozapine pellet injection. Placebo-injected age-matched mice were used as controls (Student *t* test, ** *p* < 0.01). *n* = 4 per group. B Body weight change of clozapine- or placebo-treated mice in wild-type and *Sapap3+/−* mice. There was no significant difference between clozapine- and placebo-treated mice. *n* = 5–6 per group C Survival curve of skin lesion development in *Sapap3*^-/-^ mice (left). *Sapap3*^-/-^ mice have neck and facial skin lesions (white arrows). *n* = 7–12 per group. All data are presented as means ± SEM.

**Additional file 3.** Sapap3-/- mice.

## Data Availability

All data and materials are available upon requests.

## Abbreviations

OCD: Obsessive compulsive disorder
OCS: Obsessive compulsive symptoms
WT: Wild-type
KO: Knockout
SEM: Standard error of the mean
