# Supplementary material for: Clozapine generates obsessive compulsive disorder-like behavior in mice
Source: Mol Brain. 2020 May 29;13:84. doi: 10.1186/s13041-020-00621-5 (PMC7257162; doi:10.1186/s13041-020-00621-5)
Supplement: Supplementary file 1 — Additional file 1. Raw data of the tested parameters in wild-type. [file 13041_2020_621_MOESM1_ESM.docx]

**Animals**

We used B6.129-Dlgap3^tm1^Gfng/J (008733) mice provided by the Jackson Laboratory. Exon 3 of the *Sapap3* gene was replaced with a neo cassette. The resulting mice were maintained in a C57BL/6J background and mice heterozygous for *Sapap3*-null alleles were bred to generate wild-type (*Sapap3^+/+^*), *Sapap3* heterozygous KO (*Sapap3^+/-^*), and *Sapap3* homozygous KO (*Sapapp3^-/-^*) mice. For all *in vivo* experiments, we used littermate mice. The genotypes of the offspring were determined via PCR using three primers: P1, F-5’-AGGCAATACCAACAGGGATG-3’, for the WT and *Sapap3*-null alleles; P2, R-5’-CCAGCTCTCGGAGCACAG-3’, for the *Sapap3* WT allele; and P3, R-5’-TTGATTCCCACTTTGTGGTTC-3’, for the *Sapap3*-null allele. The expected products for the WT and *Sapap3*-null alleles were 306 bp and 226 bp, respectively. Mice were housed in groups of 2–5 per cage with water and standard chow *ad libitum* in a humidity- and temperature-controlled, specific pathogen-free environment (lights on/off at 8:00 am/pm) in the Yonsei University College of Medicine Animal Care Facility. All animal experiments were approved (No. 2018-0278) by the Animal Care Committee of Yonsei University College of Medicine using US National Institutes of Health Guidelines.

**Drug**

Clozapine slow-releasing pellets (Innovative Research of America, Sarasota, FL, USA), which release clozapine at 0.02 mg/day for 60 days, were used for chronic clozapine administration. Mice at the age of 12 wk were first injected with clozapine or placebo pellets subcutaneously. Then, new pellets were regularly implanted in 60-day intervals for a total of 4 pellet implants per subject. Pellet implantation was conducted under isoflurane anesthesia using a trocar injector. After the pellet injection, the trocar injection site was sealed using the reflex skin closure system (CellPoint Scientific, Inc., Gaithersburg, MD, USA). *Sapap3^+/+^* mice at the age of 30 wk (18 wk after 1^st^ pellet injection) were used for the fluoxetine treatment experiments. Fluoxetine hydrochloride (Sigma-Aldrich, St Louis, MO) was dissolved in saline to a final concentration of 1 mg/ml. Mice were intraperitoneally injected with fluoxetine (5 mg/kg) or vehicle (saline) once a day for ten days. Behaviors were tested 24 h after the final injection.

**Grooming assessments**

Grooming behaviors of clozapine- or placebo-treated mice were analyzed at the age of 15, 20, 30 and 40 wk (3, 8, 18 and 28 wk after 1^st^ pellet injection, each). All video recording took place in the same animal behavior room in the time window from 10 am to 2 pm. Animals were allowed to acclimate to the testing room for 1 h in their home cages. Each animal was then placed in a clear acrylic chamber (30 x 23 x 18cm), which was cleaned with 70% alcohol before and after each recording. The total amount of time spent grooming in each 2-h segment was determined. Grooming included all sequences of face-wiping, scratching/rubbing of head and ears, as well as full-body grooming. Grooming bouts were episodes of grooming behavior lasting more than 3 s. All video analysis was conducted blindly and manually by two independent experimenters with an inter-observer reliability of 95%.

**Measurement of plasma clozapine concentration**

For the determination of clozapine concentrations, blood plasma samples were obtained from 12-week-old B6J mice 10 days after clozapine pellet implantation. Placebo-injected age-matched adult mice were used as controls. Concentrations of clozapine were measured using a LC-MS/MS system (AB SCIEX triple quadrupole 6500 plus, Foster City, CA, USA).

**Statistical analysis**

Data analysis was conducted with GraphPad Prism version 7 (GraphPad Software, Inc., La Jolla, CA, USA). Differences between means were assessed with a *t-*test or two-way repeated measures (RM) analysis of variance (ANOVA) as appropriate. Bonferroni *post hoc* tests were used for multiple comparisons whenever the ANOVA showed significant variance. All data are presented as means ± standard error of the mean (SEM). The significance level was set at *p* < 0.05.
