# Supplementary material for: Clozapine generates obsessive compulsive disorder-like behavior in mice
Source: Mol Brain. 2020 May 29;13:84. doi: 10.1186/s13041-020-00621-5 (PMC7257162; doi:10.1186/s13041-020-00621-5)
Supplement: Supplementary file 2 — Additional file 2: Figure S1. A Plasma clozapine concentration 10 days after clozapine pellet injection. Placebo-injected age-matched mice were used as controls (Student t test, ** p < 0.01). n = 4 per group. B Body weight change of clozapine- or placebo-treated mice in wild-type and Sapap3+/− mice. There was no significant difference between clozapine- and placebo-treated mice. n = 5–6 per group C Survival curve of skin lesion development in Sapap3-/- mice (left). Sapap3-/- mice have neck and facial skin lesions (white arrows). n = 7–12 per group. All data are presented as means ± SEM. [file 13041_2020_621_MOESM2_ESM.pdf]

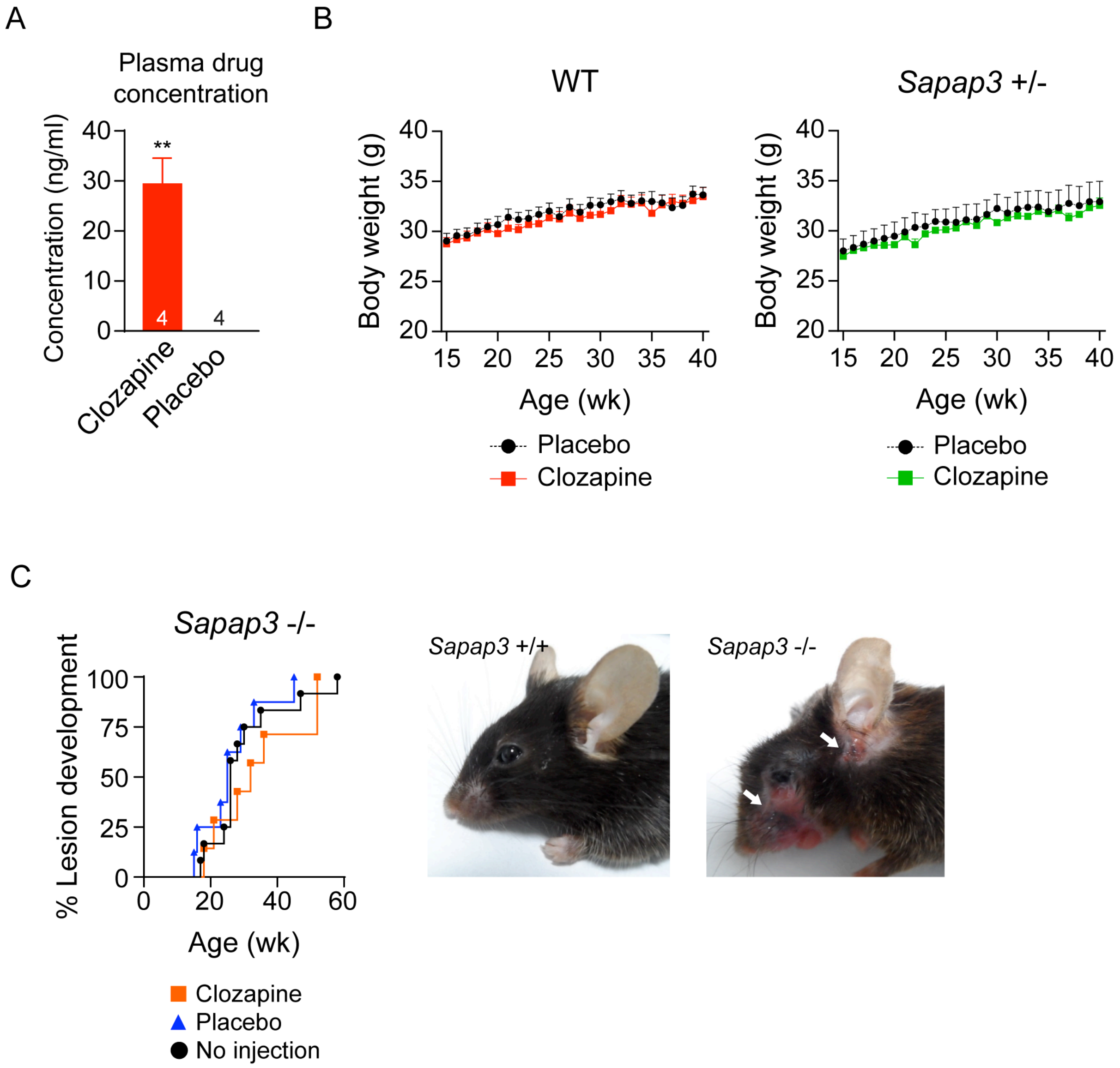

Figure S1. **A** Plasma clozapine concentration 10 days after clozapine pellet injection. Placebo-injected age-matched mice were used as controls (Student *t* test, ** $p < 0.01$). $n = 4$ per group. **B** Body weight change of clozapine- or placebo-treated mice in wild-type and *Sapap3*$^{+/-}$ mice. There was no significant difference between clozapine- and placebo-treated mice. n = 5–6 per group **C** Survival curve of skin lesion development in *Sapap3*$^{-/-}$ mice (left). *Sapap3*$^{-/-}$ mice have neck and facial skin lesions (white arrows). n = 7–12 per group. All data are presented as means ± SEM.
